# Predictive Biomarkers: A New Frontier in Bespoke Aesthetic Dermatology

**DOI:** 10.1093/asjof/ojaf155

**Published:** 2025-11-24

**Authors:** Diala Haykal, Frédéric Flament, Atchima Suwanchinda, Qian Zheng, Christopher Rowland-Payne, Anne Colonna

## Abstract

As aesthetic dermatology integrates increasingly advanced injectable and energy-based interventions, the need for biologically informed decision-making becomes critical. Complications such as granulomas, fibrosis, and post-inflammatory hyperpigmentation still affect subsets of patients due to interindividual biological variability. This commentary explores the dual role of biomarkers (measurable indicators of process, risk, or response) and hallmarks (phenotypic patterns summarizing underlying biology) in identifying patients at risk of complications, offering real-world applications such as tape stripping to evaluate our molecular patient profile. The potential of artificial intelligence to interpret complex biological signatures is discussed, along with broader implications for long-term personalization in cosmetic care. By aligning treatment protocols with skin biology, aesthetic dermatology would shift toward a predictive and preventive model, ensuring safety, precision, and durable results.

**Level of Evidence:** 5 (Therapeutic)

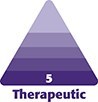

Dermatology is a broad discipline, encompassing cutaneous diseases, infections, venereology, and oncology. Within this field, aesthetic dermatology represents a specific branch dedicated to correction and optimization of skin appearance, carrying both medical responsibilities of safety and increasing societal demand. Injectable treatments and energy-based devices (EBDs) have become pillars of modern aesthetic dermatology. While their safety profiles are generally favorable, complications such as inflammatory nodules, post-inflammatory hyperpigmentation (PIH), and fibrotic tissue remodeling continue to challenge even the most experienced practitioners. These events are often unpredictable and poorly correlated with basic clinical classifications.^1^

To improve complication prevention, there is growing interest in using biomarkers, molecular signatures of inflammation, pigmentation, and fibrosis, and hallmarks, the phenotypic traits reflecting skin's biological state. When used together, they enable a shift from generalized protocols to biologically informed, patient-specific care.^2^ This approach is not only applicable to risk stratification but also extends into cosmetic personalization, including aftercare product selection and long-term skin maintenance (Table 1).

**Table 1:** Integrative Biomarker Framework for Predictive Aesthetic Care

Biological axis	Representative biomarkers/readouts	Primary sources of evidence	Clinical relevance in aesthetic skin care	Evidence base (type)
Epigenetic regulation and methylation drift	CpG methylation in COL1A1, MMP1, and ELOVL2 loci; skin-specific clocks (Horvath, VisAgeX)	Horvath et al, *Genome Biol* 2013^3^; Bienkowska et al *Front Aging* 2023^4^	Reflects intrinsic and extrinsic skin ageing; potential predictor of loss of regenerative capacity and lack of collagen remodeling	-Translational; cohort-based validation
Cellular senescence and SASP activity	p16^INK4a^, p21, IL-6, IL-8, TNF-α, MMP-1	Herranz et al, *J Clin Invest.* 2018^5^	Associated with impaired wound healing, fibrosis, and prolonged erythema after EBDs or fillers	Preclinical + observational
Chronic Inflammation and immune activation	Serum CRP, IL-17A; local IL-1α, TNF-α (tape-strip)	Franceschi et al, *Nat Rev Endocrinol.* 2018^6^	Predictive of exaggerated inflammatory responses and delayed recovery	Observational; expert consensus
Fibrotic remodeling/ECM dysregulation	TGF-β1, LOX, procollagen I/III ratio, TIMP-1	Hinz et al *Am J Pathol*. 2012^7^	Indicates susceptibility to nodule formation or hypertrophic reaction following fillers	Mechanistic; case-based
Oxidative stress and mitochondrial imbalance	ROS indices, 8-oxo-dG, SOD/CAT ratio	Poljšak et al *Acta Dermatovenerol Alp Pannonica Adriat. 2012*^8^	Correlates with pigmentary unevenness, dermal dullness, and premature photoaging	Experimental + clinical
Barrier integrity and microbiome health	Transepidermal Water Loss (TEWL), corneometry, microbial diversity index (16S rRNA)	Carrieri et al *Sci Rep*. 2021^9^	predict skin hydration, barrier resilience, and individual susceptibility to irritation and erythema following cosmetic procedures	Clinical; exploratory multicohort analysis

## DEFINITONS

Biomarkers: Quantitative indicators, molecular (eg, cytokines), cellular, structural, imaging-based, or functional (eg, barrier metrics), which reflect a biological process, risk, or response. Used for measurement and decision support.

Hallmarks (clinical/biological): Higher-order, often multiparameter phenotypes (eg, “inflammaging,” “fibrotic tendency,” “pigment-prone”) that summarize patterns across biomarkers, clinical signs, and function.

Usage rule: Biomarkers are measures; hallmarks are patterns derived from measures plus clinical context.

## FROM HALLMARKS TO ACTION: CLINICAL INTEGRATION IN PRACTICE

In modern aesthetic dermatology, there is a growing recognition that visible features such as pigmentation, textural irregularities, or scarring represent only the superficial expression of deeper biological processes. What appear clinically as solar lentigines, periorbital hyperpigmentation, or uneven post-acne pigmentation reflect underlying biological hallmarks of cutaneous dysfunction, including chronic inflammation, oxidative stress, or dysregulated melanogenesis. These hallmarks are the phenotypic outcomes of multiple underlying biomarkers, measurable molecular indicators that capture the skin's inflammatory, metabolic, and oxidative state.^10^

To move from visual impression to biological insight, the integration of biomarker analysis is essential. One increasingly accessible method is tape stripping, which allows for noninvasive sampling of the stratum corneum to assess local molecular activity.^11^ This technique can quantify cytokines such as IL-1α, IL-8, and TNF-α, along with matrix metalloproteinases (MMPs), oxidative stress markers, and melanogenesis enzymes. These biomarkers provide a snapshot of the skin's immune and metabolic status at a given moment.^12^

For instance, in patients preparing for fractional laser resurfacing, pretreatment tape stripping can reveal elevated pro-inflammatory cytokines despite clinically healthy-appearing skin. Such biological evidence suggests an increased risk of exaggerated post-procedure inflammation or PIH. In these cases, clinicians can delay treatment, lower energy settings, or introduce a pretreatment protocol with antioxidants or barrier-stabilizing agents to modulate skin reactivity.^13,14^ Similarly, patients with high oxidative stress or signs of dermal matrix degradation, detected via noninvasive biomarker sampling, may benefit from pretreatment strategies aimed at mitochondrial support, antioxidant replenishment, or MMP inhibition, depending on the molecular findings.^8, 15^

By contextualizing biological hallmarks with molecular biomarkers, clinicians can transition from generalized “skin typing” to a biologically personalized risk profile. Biological risk profiling would contribute to enhanced procedural safety by allowing practitioners to anticipate reactive or high-risk skin profiles.^16^ The articulation between visible and invisible signs, and clinical and biological hallmarks, can be further enriched by integrating the functional and dynamic dimensions of the skin. Functional parameters, including barrier integrity, immune surveillance, and biomechanical elasticity, offer critical insights into the physiological context underlying molecular hallmarks.^9^ For instance, noninvasive electrochemical techniques to assess barrier function or in vivo mechanical mapping of skin elasticity have demonstrated added value in enhancing diagnostic accuracy beyond what static imaging can provide.^17, 18^ Similarly, the analysis of facial movement patterns, such as expression-induced wrinkles or gravitational descent, reveals early indicators of tissue laxity or neuromuscular imbalance that may not be evident at rest.^19^ Incorporating these dynamic and functional aspects enables a more comprehensive assessment of cutaneous health, facilitating the anticipation of future phenotypic changes. Hallmarks should therefore be considered as temporally evolving biological signals, markers not only of the current skin state but also of its future trajectory, supporting predictive modeling and individualized strategies in both aesthetic procedures and maintenance protocols (Table 2).

**Table 2:** Translational Pathways Linking Biomarkers to Personalized Aesthetic Skin Care Strategies

Clinical context/patient profile	Underlying biological process (corresponding hallmark)	Indicative biomarkers or measurable readouts	Interpretation and biological insight	Suggested procedural strategy
Photoaged, dull, or low-collagen skin	Epigenetic dysregulation *(Hallmark: epigenetic changes)*	Acceleration of skin-specific epigenetic clocks (>5 years); increased 8-oxo-dG; reduced elasticity on cutometry	Indicates diminished regenerative potential and oxidative stress accumulation	Combine resurfacing lasers with biostimulatory fillers (PLLA, CaHA) and antioxidant preconditioning
Reactive or inflammation-prone skin	Chronic inflammaging *(Hallmark: cellular communication and chronic micro inflammation)*	Elevated IL-1α or TNF-α (tape-strip); CRP > 5 mg/L; persistent erythema	Suggests heightened inflammatory tone and delayed healing	De-escalate energy or injection density; LED or cooling-based anti-inflammatory therapy; reinforce barrier care
Fibrotic or nodular tendency	Fibroblast hyperactivation *(Hallmark: extracellular-matrix changes)*	Elevated TGF-β1 or presence of tight dermal bands	Predicts fibrosis or nodule formation risk after fillers or RF procedures	Use smaller aliquots, stage treatments, and consider ultrasound guidance; apply antifibrotic topicals post-procedure
Pigment-prone or uneven skin tone	Melanocyte hyperreactivity *(Hallmark: mitochondrial dysfunction and loss of proteostasis)*	High melanin index; increased TEWL; prior PIH	Reflects melanin overexpression and impaired antioxidant defense	Prefer longer wavelengths, pigment modulators (niacinamide, TXA), and strict photoprotection
Thin, lax, or atrophic skin	Cellular senescence *(Hallmark: stem-cell exhaustion and extracellular matrix changes)*	Increased p16^INK4a^ or SASP cytokines (IL-6, IL-8); low collagen/elastin gene expression	Indicates reduced neocollagenesis and slower tissue remodeling	Regenerative RF microneedling; collagen-stimulating injectables; extended recovery intervals
Sensitive skin with altered microbiome	Barrier dysfunction and dysbiosis *(Hallmark: microbiome alteration)*	Elevated TEWL; reduced microbial diversity index	Suggests fragile barrier and inflammation susceptibility	Begin barrier restoration and microbiome-friendly skincare 2-3 weeks before treatment
Healthy, balanced skin (low-risk)	Functional homeostasis *(Hallmark: intercellular communication)*	Normal TEWL, balanced cytokine profile, baseline elasticity	Indicates optimal regenerative potential and stable repair mechanisms	Proceed with standard treatment parameters and routine maintenance cycles

## BIOMARKERS IN ACTION: EXAMPLES FROM COSMETIC DERMATOLOGY

In filler practice, certain patients present with recurrent delayed-onset nodules despite using standardized injection techniques and materials. While mechanical trauma or infection is often suspected, the true trigger may lie in immune predisposition, reflected by elevated systemic interleukin-6 (IL-6), IL-17, or high-sensitivity C-reactive protein.

In clinics, patients with previous nodular complications are offered optional blood-based inflammatory screening. Those with heightened markers undergo lower-volume filler sessions, slower injection pacing, and product selection guided by immunogenicity profiles. This strategy has reduced our rate of recurrent nodules significantly.

Similarly, in energy-based procedures such as fractional radiofrequency or CO₂ laser, fibrotic hallmarks, including tight dermal bands, palpable induration, or a personal history of keloid or hypertrophic scarring, serve as practical warning signs of increased tissue reactivity. Clinically, these may manifest as reduced skin pliability on cutometry or focal stiffness on elasticity mapping. Their presence warrants parameter de-escalation, staged treatment sessions, and extended recovery intervals rather than invasive or routine laboratory testing. Noninvasive assessments such as cutometry, high-frequency ultrasound, or elasticity mapping generally provide sufficient evaluation in most clinics. Advanced molecular assays, such as transforming growth factor-β1 (TGF-β1) quantification or collagen-turnover profiling, remain research-level tools reserved for complex or recurrent fibrotic cases. Patients exhibiting these hallmarks benefit from cautious treatment protocols, including lower-density energy passes and the use of post-procedure fibrosis modulators such as silicone gel sheets or equivalent agents.

Epigenetic clocks estimate biological age by quantifying DNA methylation at specific CpG sites, capturing cumulative molecular alterations associated with chronological ageing and environmental exposure.^4, 20^ Three principal classes are currently recognized. The first are pan-tissue clocks, such as the original Horvath and Hannum models, which were trained across multiple human tissues and provide a general index of biological ageing.^3, 4^ The second group comprises phenotypic or mortality-trained clocks, including PhenoAge and GrimAge, which integrate physiological and mortality data and correlate more closely with systemic inflammation and disease risk.^6^ The third and most recent category encompasses tissue-specific or skin-adapted clocks, derived from keratinocyte or fibroblast methylomes, designed to improve precision in assessing cutaneous ageing processes.^21^

For example, a patient with accelerated epigenetic skin age often exhibits reduced neocollagenesis after fractional laser treatment or limited biostimulatory response to poly-L-lactic *acid* (PLLA) or calcium hydroxylapatite (CaHA) fillers. Conversely, those with a younger biological skin age tend to derive longer-lasting benefits from less intensive interventions. Thus, incorporating epigenetic age into pretreatment assessment could refine patient selection and calibrate expectations.

Pretreatment epigenetic markers should not only be used to predict response or adjust expectations. Many of these markers, especially those linked to inflammation, oxidative stress, and senescence, are biologically modifiable before any procedure. Recent evidence suggests that targeted epigenetic preconditioning can enhance procedural outcomes.^13, 15^ Examples include topical retinoids, antioxidants, niacinamide, LED photobiomodulation, microbiome-supporting skincare, and systemic interventions such as improved sleep, anti-inflammatory nutrition, senomorphic, or mitochondrial-supporting compounds. These approaches have been shown to reduce senescence-associated secretory phenotype (SASP) cytokines, improve collagen-related gene expression, and even lower skin-specific epigenetic age. Therefore, pretreatment biomarker measurement is not only valuable for risk prediction but also for identifying candidates who may benefit from biological optimization to improve healing, collagen remodeling, and overall treatment response.

Beyond procedural guidance, repeated measurement of epigenetic age over time serves as a biological marker for biological changes associated with skin interventions.^22^ As noninvasive methylation sampling and bioinformatic interpretation become more accessible, epigenetic clocks are poised to become a cornerstone in personalized aesthetic dermatology, linking visible outcomes with underlying biological resilience.

To align measurable biomarkers with the broader biological hallmarks of skin ageing, Table 2 integrates underlying mechanisms, their corresponding hallmarks, and clinical implications. This translational framework bridges bench and bedside, illustrating how biological signals can guide personalized strategies in aesthetic skin care.

## SENESCENCE-ASSOCIATED BIOMARKERS AND THEIR ROLE IN AESTHETIC RISK AND SKIN RESILIENCE

Beyond classical inflammatory or fibrotic markers, senescence-associated biomarkers represent a critical but often under-recognized component of biological skin ageing. Accumulation of senescent fibroblasts and keratinocytes in ageing skin contributes to impaired collagen synthesis, dermal atrophy, pigment dysregulation, and a pro-inflammatory microenvironment. These changes are not only responsible for visible signs of skin ageing but also increase the risk of poor healing, fibrosis, or hyperreactivity following aesthetic procedures.^5,23^

As described by Wyles et al, senescent cells exhibit a distinct secretory profile, known as the SASP, which includes elevated levels of IL-6, IL-8, MMPs, and TNF-α. These are the very cytokines and enzymes often associated with delayed complications after injectables or laser treatments.^24^ Therefore, detecting a senescent skin phenotype, via systemic markers or local assessments such as tape stripping, serves as a predictive signal for reduced procedural tolerance or altered tissue response.^13^

Integrating senescence profiling into aesthetic protocols, whether involving injectables, EBDs, or topical cosmetic care, offers a biologically informed framework that spans pre-, during-, and post-treatment care. In the pretreatment phase, identifying a senescence-associated profile through biomarkers such as IL-6, MMPs, or SASP-related cytokines can help stratify patients based on tissue reactivity, regenerative potential, and inflammatory risk. For example, a high burden of senescent fibroblasts or keratinocytes may predict suboptimal healing, persistent erythema, or complications such as delayed nodules following dermal fillers or pigmentary rebound after laser treatments. Recognizing this biological context, clinicians may opt for reduced injection volumes, lower fluence settings, or initiate preparatory skincare using barrier-repairing agents, antioxidants, or senomorphic compounds to modulate the skin microenvironment before intervention.^10,25^

In the treatment phase, senescent cells commonly exhibit diminished responsiveness to collagen-inducing therapies, such as biostimulatory fillers (PLLA, CaHA), radiofrequency microneedling, or fractional ablative lasers. Here, understanding tissue senescence can guide the choice of technique or combination strategies. For instance, pairing energy-based devices with regenerative adjuncts like platelet-rich plasma, for the biological inertia of senescent skin. Likewise, for injectable procedures, patients with senescent-prone tissue are more likely to benefit from techniques focused on soft tissue support rather than aggressive volumization.

In the post-treatment phase, senotherapeutic approaches, either topical (eg, retinoids, flavonoids) or systemic (eg, senolytics like quercetin or dasatinib), help accelerate recovery, reduce chronic low-grade inflammation, and prolong the biological benefits of the intervention.^26^ These agents are increasingly integral to maintenance skincare protocols, particularly in patients with photodamaged or biologically aged skin. Moreover, senescence profiling should inform cosmetic care selection beyond aesthetics, targeting intrinsic ageing mechanisms to complement procedural effects.

Altogether, the integration of senescence-related insights across aesthetic modalities enables a longitudinal, biology-driven model of care that goes beyond reactive treatment planning. When combined with epigenetic clocks and AI-based risk models, senescence profiling offers a more complete picture of the skin's health status, one that informs how to treat, when to treat, how to maintain, and how to preserve skin function over time. This multidimensional personalization redefines what it means to practice modern aesthetic dermatology, not only aiming for visible improvement but also for sustained, biologically harmonious skin ageing.

Beyond physiological diagnostics and complication prevention, the incorporation of biological hallmarks into aesthetic practice may also contribute to improved psychological well-being and perceived treatment satisfaction. Studies suggest that enhanced awareness of one's skin type, sensitivity, and biological profile can foster a sense of control and trust in the therapeutic process, leading to greater self-confidence and reduced anxiety surrounding procedures.^27, 28^ Additionally, interventions that combine visible skin improvements with subjective feelings of care and personalization, such as daily self-massage or tailored at-home regimens, have been shown to positively influence mood, reduce stress, and support emotional well-being. As biological personalization evolves, understanding its role in the psychodermatological dimension of care becomes increasingly relevant.^28,29^ Hallmark-guided protocols may not only optimize biological outcomes but also reinforce patient satisfaction by addressing the emotional expectations and experiences surrounding aesthetic interventions.

## Artificial Intelligence AND MACHINE LEARNING: INTEGRATING MULTIMODAL DATA FOR PREDICTIVE PRECISION

The integration of biomarkers and hallmarks into clinical practice creates a data-rich environment that challenges traditional pattern recognition. Artificial intelligence (AI), particularly machine learning (ML) algorithms, can assist in processing large, heterogeneous datasets by identifying patterns that may not be visible through classical statistics.^30-33^

In addition to in-clinic diagnostics, AI-driven strategies now support personalized coaching and home-based follow-up before and after aesthetic interventions. Following the initial assessment of biological hallmarks, patients can benefit from connected tools, such as mobile applications, wearable biosensors, and AI-integrated skin imaging, which enable continuous monitoring of recovery progress and environmental exposures. These technologies allow for the dynamic adjustment of skincare routines, taking into account daily fluctuations in skin condition, humidity, UV index, and healing biomarkers. Beyond topical modifications, the personalization of recovery can also extend to the selection of home-use devices, such as LED systems, microcurrent applicators, or cryo-based tools, matched to the patient's specific biological needs during the repair phase. This hybrid model, linking clinic-based diagnostics with smart home-based interventions, has been recently conceptualized as a new frontier in dermatological care.^34^ By extending biological personalization beyond the consultation room, practitioners can support tissue regeneration more effectively and enhance both safety and satisfaction across the continuum of care.

For example, combining patient-reported history, visual hallmarks (eg, texture, erythema, pigmentation), and quantified biomarkers (inflammatory cytokines, fibrotic mediators, pigmentary enzymes) allows ML models to generate risk scores for filler-related granulomas or PIH after EBDs.^7,35^ Algorithms trained on retrospective complication cases can predict future risks with increasing accuracy. Moreover, AI can be used to dynamically update risk stratification. A patient who begins with low-risk markers may shift into a reactive state post-treatment, detected by elevated oxidative markers or epidermal stress signals. These changes can be captured using smart skin diagnostics or wearables, allowing clinicians to adjust aftercare in real-time.^36^

In research settings, AI is already being used to match specific biomarker constellations with best treatment outcomes, helping to establish “biological pathways to beauty.” The integration of AI into routine aesthetic care will allow simultaneous interpretation of visual, structural, and molecular skin data.

## BEYOND COMPLICATIONS: PERSONALIZATION IN COSMETIC AFTERCARE

The relevance of biomarkers and hallmarks extends beyond predicting complications. They also play a role in customizing skincare regimens, maintenance protocols, and retreatment timelines. For example, patients with chronic oxidative stress benefit from prolonged antioxidant use even after successful EBD sessions. Others with pigmentary dysregulation often require long-term maintenance with stabilized tyrosinase inhibitors or DNA repair enzymes, even in the absence of visible lesions.^37^

Moreover, hallmarks such as slow healing, prolonged erythema, or fragile capillary networks indicate a need to avoid aggressive treatments like deep phenol peels or ablative CO₂ lasers, not just because of risk, but because of a reduced likelihood of sustained benefit. Conversely, individuals with balanced inflammation-resorption signatures may be ideal candidates for regenerative procedures, including platelet-rich plasma, exosomes, or controlled mechanical injury.^38^

Biological personalization also opens avenues for home-based cosmetic care. For example, skin care lines could be matched not to phototype or age, but to biomarker profiles: a “Type 1 skin” with high TGF-β1 and low MMPs might receive matrix-preserving peptides, while a “Type 2” profile with upregulated tyrosinase expression (TYR) and oxidative burden might benefit from pigment modulators and polyphenols.

### Practicability

Translating biomarker- and hallmark-based personalization into everyday aesthetic practice requires a pragmatic and stepwise approach. Although current assays for inflammatory or fibrotic markers remain largely confined to research settings, several techniques, such as tape stripping, transepidermal water loss measurement, or skin elasticity mapping, are already feasible in clinics and can serve as entry points for biological profiling. Integrating these noninvasive diagnostics within pretreatment assessments or follow-up visits adds minimal procedural time while providing actionable insights for protocol adjustment. The progressive inclusion of AI-assisted image interpretation and simplified molecular panels will further facilitate accessibility, allowing practitioners without laboratory infrastructure to benefit from biologically informed decision support. Ultimately, the practicability of this model lies in its scalability: beginning with simple, measurable indicators of barrier integrity or inflammation today, and evolving toward comprehensive multi-omic and digital analyses as technology and cost-efficiency advance.

## CONCLUSIONS

The integration of biomarkers and hallmarks in aesthetic dermatology signifies a transition toward biologically anchored personalization. Complication prevention, while crucial, is only the first frontier. Although preventive strategies are already implemented in current clinical practice, the systematic integration of biomarkers and biological signatures opens the way to a higher level of personalization and anticipation.

By systematically evaluating inflammatory status, fibrotic risk, pigmentary tendencies, and even epigenetic age, clinicians can refine treatment selection, timing, and intensity, not just to avoid adverse effects, but to optimize biological harmony with every intervention. This level of personalization empowers practitioners to consider not only the visible needs of the skin but also its molecular vulnerabilities, regenerative reserves, and responsiveness over time. As techniques such as tape stripping, AI-based imaging, and predictive analytics become more accessible and better validated, the barrier to integrating biomolecular diagnostics into aesthetic workflows will continue to fall. These innovations will also enable real-time monitoring of skin health, opening new possibilities for adjusting protocols dynamically, based on measurable biological response rather than subjective assessment alone.

Ultimately, this evolution will redefine aesthetic excellence, not merely as the absence of complications or short-term improvement, but as the presence of biologically harmonious, lasting, and individualized outcomes. A future where cosmetic care respects and responds to each patient's molecular signature is no longer speculative, it is imminent. Embracing this paradigm is not only a scientific necessity but also a clinical opportunity to elevate the standard of care in aesthetics toward a truly regenerative and preventive discipline.
